# Scutellarin ameliorates neonatal hypoxic-ischemic encephalopathy associated with GAP43-dependent signaling pathway

**DOI:** 10.1186/s13020-021-00517-z

**Published:** 2021-10-18

**Authors:** Rui-Ze Niu, Liu-Lin Xiong, Hao-Li Zhou, Lu-Lu Xue, Qing-Jie Xia, Zheng Ma, Yuan Jin, Li Chen, Ya Jiang, Ting-Hua Wang, Jia Liu

**Affiliations:** 1 Animal Zoology Department, Kunming Medical University, Kunming, 650031 China; 2 Institute of Neurological Disease, Translational Neuroscience Center, West China Hospital, Sichuan University, Chengdu, 610041 China; 3 Institute of Neuroscience, Kunming Medical University, Kunming, 650031 China; 4 Department of Anesthesiology, Affiliated Hospital of Zunyi Medical University, Zunyi, 550000 China; 5 The Clinical Hospital of Chengdu Brain Science Institute, MOE Key Lab for Neuroinformation, University of Electronic Science and Technology of China, Chengdu, 610054 China

**Keywords:** Scutellarin, Hypoxic-ischemic encephalopathy, GAP43, Neuroprotection

## Abstract

**Background:**

Neonatal hypoxic-ischemic encephalopathy (HIE) refers to the perinatal asphyxia caused by the cerebral hypoxic-ischemic injury. The current study was aimed at investigating the therapeutic efficacy of Scutellarin (Scu) administration on neurological impairments induced by hypoxic-ischemic injury and exploring the underlying mechanisms.

**Methods:**

Primary cortical neurons were cultured and subjected to oxygen–glucose deprivation (OGD), and then treated with Scu administration. The growth status of neurons was observed by immunofluorescence staining of TUJ1 and TUNEL. Besides, the mRNA level of growth-associated protein 43 (GAP43) in OGD neurons with Scu treatment was detected by quantitative real-time polymerase chain reaction (qRT-PCR). To further verify the role of GAP43 in Scu treatment, GAP43 siRNA and knockout were applied in vitro and in vivo. Moreover, behavioral evaluations were performed to elucidate the function of GAP43 in the Scu-ameliorated long-term neurological impairments caused by HI insult. The underlying biological mechanism of Scu treatment was further elucidated via network pharmacological analysis. Finally, the interactive genes with GAP43 were identified by Gene MANIA and further validated by qRT-PCR.

**Results:**

Our data demonstrated that Scu treatment increased the number of neurons and axon growth, and suppressed cell apoptosis in vitro. And the expression of GAP43 was downregulated after OGD, but reversed by Scu administration. Besides, GAP43 silencing aggravated the Scu-ameliorated neuronal death and axonal damage. Meanwhile, GAP43 knockout enlarged brain infarct area and deteriorated the cognitive and motor dysfunctions of HI rats. Further, network pharmacological analysis revealed the drug targets of Scu participated in such biological processes as neuronal death and regulation of neuronal death, and apoptosis-related pathways. GAP43 exhibited close relationship with PTN, JAK2 and STAT3, and GAP43 silencing upregulated the levels of PTN, JAK2 and STAT3.

**Conclusions:**

Collectively, our findings revealed Scu treatment attenuated long-term neurological impairments after HI by suppressing neuronal death and enhancing neurite elongation through GAP43-dependent pathway. The crucial role of Scutellarin in neuroprotection provided a novel possible therapeutic agent for the treatment of neonatal HIE.

**Graphic abstract:**

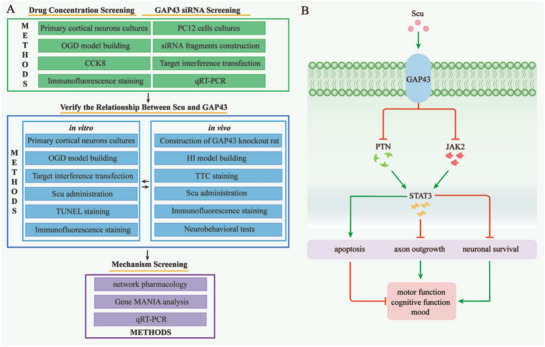

**Supplementary Information:**

The online version contains supplementary material available at 10.1186/s13020-021-00517-z.

## Background

Neonatal hypoxic-ischemic encephalopathy (HIE) has been considered one of the leading causes in neonatal death due to its devastating impact on neonatal brain development [1]. It’s reported that 15–20% of affected newborns (1–3 per 1000 full term births) will die in the postnatal period, and an additional 25% will develop severe and irreversible neuropsychological sequelae [2]. Accumulating evidence confirmed that HIE could bring about acute injuries, such as reperfusion injury, edema, increased intracranial pressure, impaired autoregulation, and hemorrhage, which are known as important pathologies of later neurodevelopmental impairments, such as epilepsy, behavioral deficits, learning disorders, and cerebral palsy (CP) [3]. Presently, the treatment of HIE prevailingly tends to control cerebral palsy, brain edema, and long-term complications [4], such as fluids therapy [5], antiseizure medication [6], stem cell therapy [7], and hypothermia [8]. Although hypothermia has been viewed as the standard care for newborns with HIE, the efficacy for alleviating long-term impairments remains modest and the benefits for infants with severe HIE have been restricted. Accordingly, the underlying mechanisms involved in the process of HIE and more effective therapeutic methods need to be further investigated.

Scutellarin, extracted from the dried Erigeron, is an active ingredient of flavonoid glucosides [9]. Scu has been previously reported to be capable of expanding blood vessels, reducing vascular resistance, improving microcirculation and inhibiting aggregation of platelets [10]. Besides, Scu exhibited a number of cardio- and neuroprotective effects by inhibiting p38 activity [11], ERK phosphorylation, and TGF-β expression and fibrosis [12]. The compound could reduce infarct volume and alleviate neurological deficits by increasing eNOS expression while inhibiting the induction of iNOS [13]. The neuroprotective effects of Scu on middle cerebral artery occlusion (MCAO)-induced brain damage in rats has been widely investigated, and the results revealed that Scu treatment improved the neurological score, diminished the percentage of brain infarct volume, and enhanced endogenous antioxidant activity [14]. Another study indicated that the neuroprotective effects of Scu on attenuating brain ischemic injury-induced apoptosis might be associated with inhibition of PARP-dependent mitochondrial dysfunction and subsequent translocation of apoptosis-inducing factors (AIF) [15]. However, little is understood about the role of Scu in HIE-induced long-term complications as well as the underlying mechanism. Thus, it is extremely urgent to investigate the efficacy of Scu in HI-induced long-term dysfunctions and explore the underlying mechanism.

Growth-associated protein, GAP43, a component of growth cones and a nervous tissue-specific cytoplasmic protein that can be attached to the membrane via a dual palmitoylation sequence on cysteines 3 and 4, is induced when neurons extended axons [16]. This phosphoprotein is neuron-specific and expressed in neuronal somata, axons, and growth cones during pre- and early postnatal development [17]. GAP43 has been considered to play a crucial part in neuronal growth, axonal regeneration and stabilization of synaptic function [17]. Inhibiting GAP43 expression exerts adverse effects on axon outgrowth [18]. Previous studies on homozygous GAP43 knockout rat’s line found it lethal for neonatal rats because GAP43 plays a key role in the development of the mammalian central nervous system [19]. A recent study reported GAP43 as a novel metastasis promoter in non-small cell lung cancer [20] but GAP43 expression is usually highest during critical periods of neural system development [21]. Based on the above-mentioned crucial function of Scu and GAP43, we investigated whether a solid association can be found between Scu treatment and GAP43 expression in the treatment of HIE.

## Methods

### Animals care

Timed pregnant female Sprague–Dawley (SD) rats were purchased from Animal Centre of Kunming Medical University and housed in individual cages under a 12-hour (h) light/dark cycle, with food and water available throughout the study. Seven-day-old postnatal pups were fed for the following experiments. The animal study was legally approved by the Animal Care & Welfare Committee of Kunming Medical University with the approval number: 2015-1A. All experiments conformed to the Guide for the Care and Use of Laboratory Animals published by the US National Institutes of Health. Rats were randomly arranged to the four groups for behavioral tests (n = 5/group): HI group (rats were subjected to HI insult), Sham group (rats were subjected to exposure of the right carotid artery only), HI + SCU + GAP43^+/+^ group (HI rats received SCU administration), HI + SCU + GAP43^−/−^ group (GAP43-knockout rats were subjected to HI insult and received Scu administration), and the two groups for TTC staining (n = 5/group): HI-GAP43^+/+^ group (rats were subjected to HI insult) and HI-GAP43^−/−^ group (GAP43-knockout rats were subjected to HI insult). Twenty 1-day-old newborn rats were used for the collection and cultures of primary cortical neurons.

### Primary cortical neurons cultures

The culture of primary cortical neurons was carried out as previously described [22]. Briefly, the cerebral cortexes of 1-day-old neonatal rats were harvested, homogenated and culture. The complete culture medium composed of DMEM/HIGH GLUCOSE, 10% fetal calf serum and 1% penicillin–streptomycin solution as used for cell culture. Neurons were plated in 6-well plates (Corning, United States) coated with poly-d-lysine and laminin (Sigma-Aldrich, St. Louis, MO, United States) at a density of 2–5 × 10^5^/ml, and incubated at 37 °C, 5% CO_2_. Moreover, the complete medium was replaced by neuron specific medium (neuron basal + 2% B27, no serum) (Invitrogen, Carlsbad, CA). The culture medium was then changed the next day. Neurons were divided into 4 groups (n = 3 well/group): normal group (neurons without any treatment); OGD group (Neurons were subjected to OGD); Control group (OGD neurons received 1/3000 DMSO); OGD + Scu 3 μM group (OGD neurons received 3 μM Scu administration).

### Oxygen–glucose deprivation model

Cultured primary cortical neurons for 5 days were firstly washed with PBS for three times and then placed into the glucose-free medium (Gibco, USA) at 37 °C. Subsequently, the cells were transferred into a hypoxia chamber equipped with a compact oxygen controller to maintain the inner concentration of 95% N_2_, and 5% CO_2_ at 37 °C for 2 h. Afterwards, these obtained cells were put back into normal DMEM medium with 95% air and 5% CO_2,_ and incubated for 24 h for reoxygenation.

### Scu administration in vitro

Firstly, 150 mg Scu (Batch no.20200602, Longjing Tech, Kunming, China), after being exposed under ultraviolet irradiation for half an hour, were dissolved by 1 ml DMSO into 150 mg/ml and then implanted into the previously cultured normal primary cortical neurons and neurons post OGD. As for vehicle treatment, the equivalent 1/3000 DMSO was added into the normal group and OGD group. SiRNA of GAP43 was performed to verify the role of GAP43 expression on HI injury. In each group, cells were observed at 24 h, 48 h and 72 h respectively after drug administration to monitor the survival of cells.

### Stimulation ratio measurement

Cell counting kit-8 (CCK-8) was used to detect the 50% effective concentration (EC50) of primary cortical neurons under Scu administration at concentrations of 0.1 μM, 0.2 μM, 0.5 μM, 1 μM, 3 μM, 10 μM, 15 μM, 50 μM, 100 μM. Following Primary cortical neurons were cultured for 7 days, OGD was performed and then neurons (1 × 10^5^ per well) were seeded into 96-well plate in a total volume of 100 μl medium containing 10% FBS (Hyclone, USA) and 1% PBS (Hyclone, USA). Twenty-four h after culturing, Scu was added into cells for co-incubation. CCK8 solution was subsequently added into a tri-gas incubator for 4 h. Twenty-four h later, the spectrometric absorbance at 490 nm was measured by a microplate reader (Model 680; Bio-199 Rad, Hercules, CA, USA). All the procedures were performed in triplicate and repeated at least three times.

### Immunofluorescence staining

Immunofluorescence staining was performed to observe the status of damaged neurons with Scu administration. Briefly, at 24 h post OGD, the cells in 96-well plate were washed three times with 0.01 M PBS and fixed using 4% paraformaldehyde for 10 minutes (min), followed by subsequent washes with 0.01 M PBS. Then, the cells were blocked with 0.1% sodium citrate and 5% horse-goat serum plus 0.3% TritonX-100 prior to incubation overnight at 4 °C with primary antibodies (Tuj1, mouse, 1:200, Abbkine). After washing three times with 0.1 M PBS (pH 7.4) containing 0.1% Tween 20 for 5 min each time, sections were incubated for 1 h at 37 °C with secondary antibody labeled with fluorescent dyes (anti-mouse, Abbkine, 1:100). Finally, images were taken under a fluorescence microscope and the number of positive cells of Tuj1 was observed.

### Terminal-deoxynucleoitidyl Transferase Mediated Nick End Labeling (TUNEL) staining

TUNEL staining was employed to detect neuronal apoptosis. Sections were fixed with 4% paraformaldehyde for 10 min after rinsed three times with PBS for 5 min each time. Subsequently, being rinsed with PBS again, sections were sealed with PBS containing 0.1% Triton in the ice bath for 2 min. The TUNEL reaction mixture was prepared in the dark: enzyme solution and label solution in a ratio of 1:9 on ice. The specimens were then put into a dark box to incubate at 37 °C for 1 h, and DAPI containing anti-fluorescence quencher were added to stain the cells which were incubated for 3 min at room temperature. A high-content cell imaging system was finally used for picture collection. Positive cells were counted by two researchers blinded to the experiment.

### Real-time quantitative polymerase chains reaction (RT-qPCR)

The total RNA fraction was extracted using Trizol reagent (Takara Bio Inc., Otsu, Japan) and reverse transcribed using RevertAid™ First Strand cDNA Synthesis System (Invitrogen). Quantitative PCR reactions were carried out with Power SYBR (DBI Bioscience) according to the manufacturer’s instructions. The expression level of each gene was normalized to that of GAPDH using the 2^−ΔΔct^ method. Primers used for the reactions are as follows:

GAPDH: forward: TGACTTCAACAGCGACAC CCA,

GAPDH: reverse: CACCCTGTTGCTGTAGC CAAA;

GAP43: forward: TGTT GCCGATGGGGTGGAGA,

GAP43: reverse: CCGTTGGAGGCTGGGCTGTT;

PTN: forward: CAGTGGAGTGTGTGTGTGCC;

PTN: reverse: GATTCTGCTTGAGGTT TGGG;

STAT3: forward: GACAAAGACTCTGGGGACG,

STAT3: reverse: ATTG GGGGCTTGGTAAAAA;

JAK2: forward: CGGCTGGGCAGTGGAGAGT,

JAK2: reverse: CGGTGATGGTGCGATTTGG.

### PC12 cells cultures

PC12 cell line, purchased from ATCC (ATCC CRL-1721), were maintained in Dulbecco’s modified Eagle’s medium composed of 0.11 g/l sodium-pyruvate, pyridoxine, 10% horse serum, 5% tetracycline-free fetal calf serum, 10 mM l-glutamine, penicillin/streptomycin (100 units/ml and 100 μg/ml, respectively) and 100 μg/ml geneticin. Cells were seeded out at a density of 1.5 × 10^4^ cells/cm^2^ and incubated at 37 °C with 5% CO_2_ at 90% humidity. The culture medium was changed every 3 days.

### Screening of effective siRNA sequences

Two siRNA sequences and a random control sequence, purchased from Guangzhou Ruibo Company (Guangzhou, China), were designed by referring to the gene database of GAP 43 from NCBI. In short, as PC12 cells grew to 40% confluence, fresh medium containing siRNA fragments was added to cells. Afterward, PC12 cells were randomly divided into the normal group, NC group, Reagent group, F1 group, and F2 group. Moreover, qRT-PCR was employed to verify the interference efficiency 48 h later, and the most efficient siRNA fragment was opted out for the following experiment.

### Target interference transfection

After being cultured for 5 days, primary cortical neurons post OGD were randomly divided into 5 groups as described above. Then half of the culture medium was removed before 60 μl 1X buffer, 5 μl siRNA and 5 μl siRNA reagent were added to each well of 6-well plates. Afterward, the neurons were incubated at room temperature for 15–30 min with the addition of 1 ml medium per well 24 h later. After 48 h, the morphology and number of neurons were observed from Leica AF6000 cell station by choosing 5 randomly selected high power fields (200X). The interference efficiency was determined using RT-qPCR following transfection for 4 days.

### HI model establishment

A hypoxic-ischemic model of HIE was generated as previously described [23]. Briefly, the 7-day newborn SD rats (weighing 12–15 g, both sexes) were anesthetized with isoflurane (4% for induction, 2% for sustained inhalation anesthesia) and were used for HI model establishment, which were subjected to ligation of right common carotid artery and 2-h hypoxia in an airtight chamber containing 8% O_2_ and 92% N_2_. Rats in the sham group were only treated with exposure of the right carotid artery.

### Triphenyl tetrazolium chloride (TTC) staining and evaluation of infarction volume

At 24 h after HI, rats were euthanatized under deep anesthesia with 4% isoflurane inhalation for 2 min. After the consciousness of the rats was evaluated by claws clamping, brains were rapidly harvested and sectioned into 2 mm coronal sections in a rat brain matrix (Seino Co., Ltd. Beijing, China) before being immersed in 2% solution of 2,3,5 triphenyl tetrazolium chloride (Sigma Co., St Louis, MO, USA). Afterward, all slices were placed into the incubation chamber at 37 °C for 30 min. Following being fixed by paraformaldehyde, the infarct site was tracked and analyzed by Image J Software (Version 1.43u; National Institutes of Health, Bethesda, MD, USA). Infarct percentage = (infarct area/the whole brain area) × 100%. Brain swelling = the ipsilateral hemisphere area − the contralateral hemisphere area.

### Scu administration in vivo

Firstly, 1 g Scu (Batch no.20200602, Longjing Tech, Kunming, China) was dissolved by DMSO into 120 mg/ml after being exposed under ultraviolet irradiation for half an hour, and then diluted into 4 mg/ml by 0.9% normal saline. Subsequently, Scu of 20 mg/kg was administered intraperitoneally at 24 h after HI. The control groups were intraperitoneally injected with the same dose of 1/3000 DMSO. The injection lasted for two weeks. Neurobehavioral scores were performed at 8 weeks after the operation.

### Network pharmacological analysis

Traditional Chinese Medicine System Pharmacology Database (TCMSP, http://tcmspnw.com/) was applied for the drug targets of Scu. GO enrichment analysis provides all GO terms that are significantly enriched in targets compared to the genome background and filters the targets that correspond to biological functions. All the targets were mapped into GO terms in the Gene Ontology database (http://www.geneontology.org/), gene numbers were calculated for every term. Pathway enrichment analysis identified significantly signal transduction pathways in targets in the KEGG pathway database (http://www.genome.jp/kegg/). In this study, R software version 3.6.1 (http://www.r-project.org) with several R packages, such as clusterProfiler, org.Hs.eg.db, enrichplot, and ggplot2, was applied to draw the barplot, bubble diagram, and signalling pathway map during GO and KEGG enrichment analysis. The statistics were collected by the ClueGO and CluePedia plugins with the False Discovery Rate (FDR) Correction set as ≤ 0.05 The R packages are available on Bioconductor (https://www.bioconductor.org/).

### Construction of GAP43 knockout rat

To confirm the function of GAP43 in HIE, CRISPER/CAS9 technology was applied to constructed GAP43 KO rats which were produced and provided by Cyagen Company (Guangzhou, China). Two targets were designed for GAP43, and two pairs of oligonucleotide chains (TGGGAGAGGTATATCCGGAA and GCTGCCTGG AGACACATCGA) were synthesized to prepare single gRNA. Then, more KO rats were reproduced by mating and detected by genotype identification.

### Genotype identification

For neonatal rats aging 3–5 days, the toes and tail tips were collected and numbered. Then, rats’ genomic DNA was extracted using Transgen's genomic DNA extraction kit (ee101-12), and PCR detection was performed with the amplification primer: Rat GAP43 forward: TGTTGCCGATGGGGTGGAGA, Rat GAP43 reverse: CCGTTGG AGGCTGGGCTGTT. In addition, the PCR amplification reaction system was as follows: a mixture with 10 µl PCR master mix, 0.6 µl upstream primers, 0.6 µl downstream, 3 µl DNA template and 5.8 µl water. The thermal cycling conditions were performed as: initial denaturation at 94 °C for 5 min, and 35 cycles of denaturation at 94 °C for 30 s, annealing at 59 °C for 30 s, with elongation at 72 °C for 30 s, followed by elongation at 72 °C for 5 min and the storage at 12 °C. Subsequently, agarose gel electrophoresis system was applied to visualize the final genotype detection under U.V after 55 min electrophoresis at 150 V.

### Neurobehavioral tests

Zea-longa score test was performed daily after HI for 2 weeks to verify the successful establishment of the HI model [24]. In terms of long-term neurological function, Morris water maze, Y-maze test, open field test and Rotarod test were performed at 8 weeks post HI as described in our previous study [22].

### Bioinformatics prediction

GeneMANIA (http://genemania.org), known as a flexible user-friendly web site for single gene queries, multiple gene queries and network search, has been extensively used to generate hypotheses about gene function, analyze gene lists and prioritize genes for functional assays [25]. To further determine the potential network related to GAP43, GeneMania (http://genemania.org/) was applied in this study. The detailed connections among GAP43 and related genes will be returned after query.

### Statistical analysis

Experimental data were analyzed by SPSS 17.0 software. Graphs were generated using Graphpad Prism 6. For comparing multiple groups, a one-way analysis of variance with post hoc Tukey’s test was used. The data between two groups were analyzed by two-tailed Student's t-test. The water maze data were analyzed by two-way analysis of variance with Dunnett’s multiple comparisons test. All the experimental animals and cells were randomly grouped, and the samples were normally distributed. Tukey post-Hoc test was used when assuming homogeneity of variance, otherwise Dunnett’s T3 test was used instead. Differences were considered statistically significant when *p* < 0.05, and the significance level is shown by *p < 0.05; **p < 0.01; ***p < 0.001.

## Results

### Scu treatment promoted neuronal growth post OGD

**Fig. 1:**
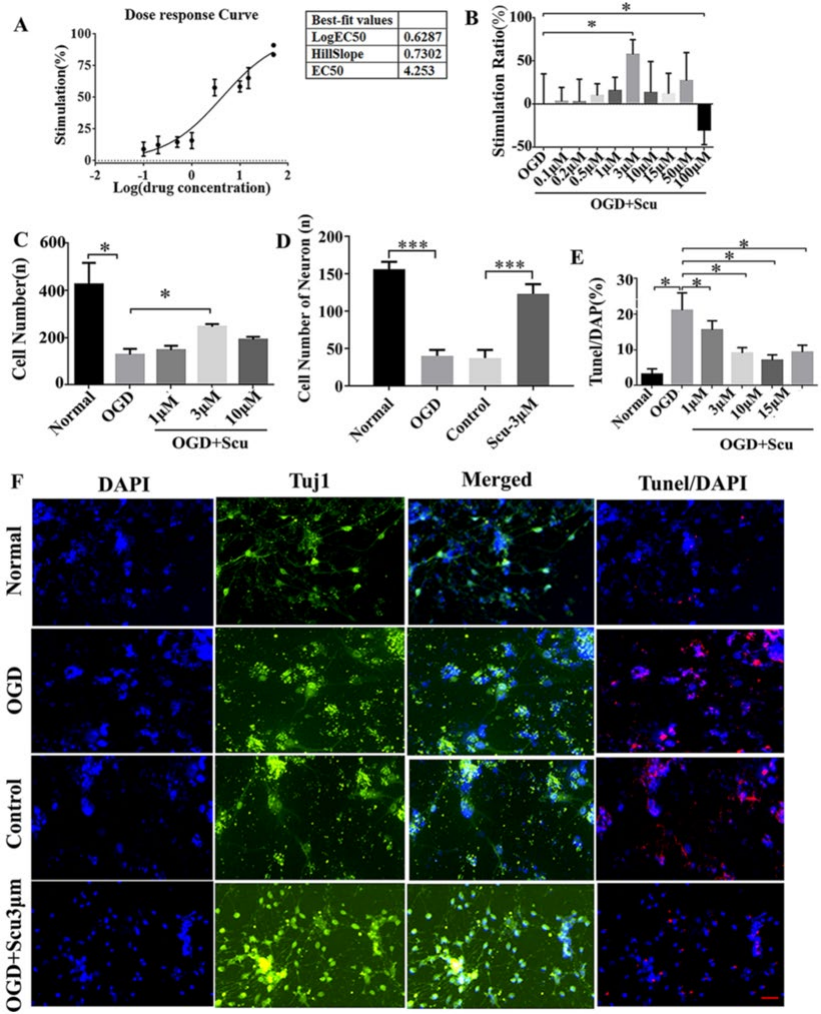
Effects of Scu administration on primary cortical neurons post OGD. **a** The dose response curve showed the significant variation from log (EC50) (− 2) to log (EC50) 2. **b**, **c** Stimulation ratio comparison among the Scu groups of different concentrations, normal group, and OGD group. There was a statistically significant difference in Scu 3 μM group and Scu 10 μM group compared with the normal group and OGD group. **d** Cell number among the normal, OGD, Scu 1 μM, Scu 3 μM and Scu 10 μM groups. **e** TUNEL/DAPI results showed cell apoptosis in the normal, OGD, OGD + Scu 1 μM, OGD + Scu 3 μM, OGD + Scu 10 μM and OGD + Scu 15 μM groups. **f** Immunofluorescence staining results of Tuj1 in the normal, OGD, Control and OGD + Scu 3 μM groups. *Normal* only cell culture group, *OGD* Oxygen–glucose deprivation, *Control* OGD + DMSO group, *Scu* Scutellarin, *TUNEL* Terminal-deoxynucleoitidyl Transferase Mediated Nick End Labeling, *DAPI* 4ʹ,6-diamidino-2-phenylindole, a fluorescent dye used in nucleic acid stain. *p < 0.05, scale bar = 50 μm, n = 3/group

One day after OGD insult, neurons were treated with Scu, followed by subsequent CCK8 assay. In accordance with dose–response curve analysis, the best-fit EC50 value for Scu was 4.253 μM (Fig. 1a). To determine the optimal Scu concentration for primary cortical neurons post OGD, we treated neurons with Scu of different concentrations and found that the stimulation ratio was notably increased in Scu 3 μM group and Scu 10 μM group, especially in the Scu 3 μM group (Fig. 1b, p < 0.05). Relative to the OGD group, the cell number was obviously increased in the Scu 3 μM group while Scu 10 μM showed inhibitory effect (Fig. 1c, p < 0.05). Additionally, the number of neurons in the OGD group was significantly reduced in comparison to the normal group and cell number in the Scu 3 μM group was increased compared to the OGD group (Fig. 1d, p < 0.05). Furthermore, significant differences were observed in the normal, OGD, OGD + Scu 1 μM, 3 μM, 10 μM and 15 μM groups after TUNEL/DAPI staining. Apoptotic cells were largely decreased in Scu 3 μM and Scu 10 μM groups compared to the OGD group (Fig. 1e, p < 0.05). Thus, OGD neurons were treated with Scu 3 μM in the following experiments. The outcomes of immunofluorescence staining showed the number of Tuj1^+^ cells and axon length decreased significantly in the OGD group compared to the normal group (Fig. 1d, f, p < 0.001). There was no significant difference between the Control group (OGD + drugs solvent group) and OGD group about the number of Tuj1^+^ cells. The Tuj1^+^ cells were significantly larger and more in the OGD + Scu 3 μM group than in the Control group (Fig. 1d, f, p < 0.001). Additionally, the apoptotic cells in the OGD + Scu 3 μM group exhibited obvious reduction relative to that in the OGD group (Fig. 1e, f, p < 0.05).

### GAP43 expression was effectively interfered by siRNA transfection

**Fig. 2:**
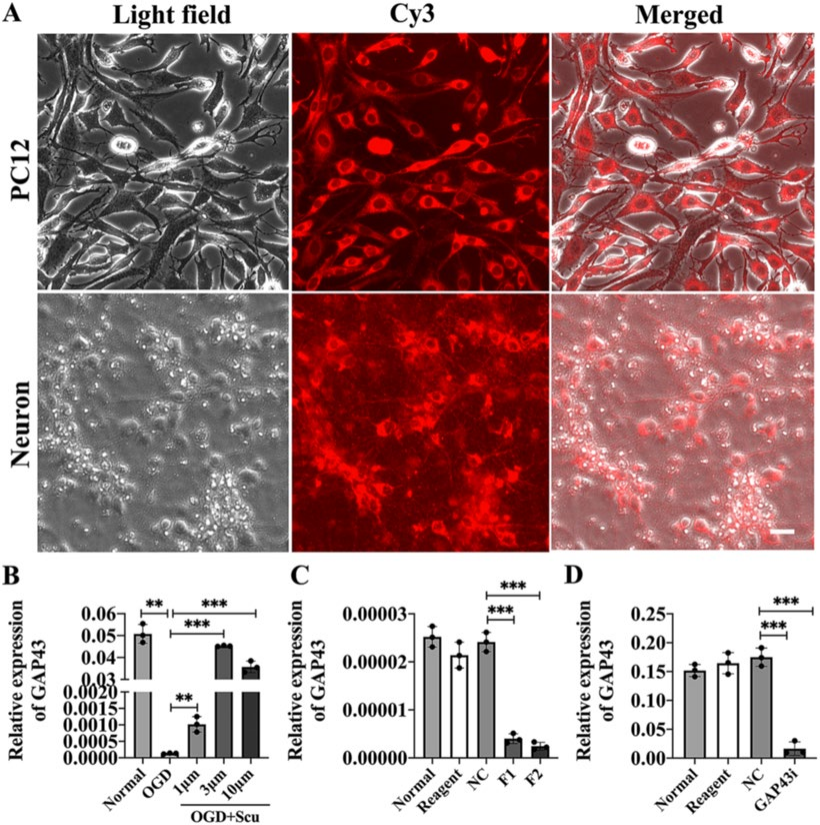
The siRNA transfection to silence the expression of GAP43. **a** PC12 cells and primary cortical neurons were transfected by CY3 (red) labeled GAP43-si then visualized by fluorescent microscope. **b** The relative mRNA expression changes of GAP43 in neurons before and after Scu administration detected by qRT-PCR. **c** The relative mRNA levels of GAP43 after transfection in the normal, reagent, NC, F1, F2 groups detected by qRT-PCR. **d** The relative mRNA expression variation of GAP43 after interference in the normal, Reagent, NC and GAP43i groups detected by qRT-PCR. The cells in the reagent group were treated with transfection reagents (without siRNA), and the cells in the NC group were treated with an irregular small RNA consistent with the sequence base number of RNAi but random sequence. *OGD* Oxygen–glucose deprivation, *RT-qPCR* real-Time quantified polymerase chain reaction, *Scu* Scutellarin, *NC* negative control, *F1* Fragment 1, *F2* Fragment 2, *GAP43i* GAP43 silencing. Data are presented as the mean ± SD. *p < 0.01, *p* < 0.001, scale bar = 50 μm, n = 3/group

In order to observe the effect of Scu treatment on the expression changes of GAP43 in OGD neurons, RT-qPCR was performed at 48 h after Scu administration. The results showed that the relative mRNA expression of GAP43 was sharply reduced in OGD group relative to normal group (*p* < 0.01), but its expression was significantly reversed after Scu treatment indicated by the obvious elevated expression in OGD + Scu 1 μM, OGD + Scu 3 μM and OGD + Scu 10 μM groups compared with that in the OGD group (Fig. 2b, p < 0.01, *p* < *0.001*). We further employed siRNA to verify the role of GAP43 in vitro*.* The effectiveness of candidate siRNA sequences F1 and F2 on silencing GAP43 expression was detected. QRT-PCR results showed that F1 and F2 siRNA sequences could decrease the expression of GAP43 in contrast to NC group, and the F2 group was more effective than F1 group (Fig. 2c, p < 0.001). After transfection, histological results demonstrated CY3-labeled siRNAs were successfully transfected into PC12 cells and primary cortical neurons (Fig. 2a), and GAP43 was seldom expressed in neurons after siRNA transfection (Fig. 2d, p < 0.001).

### GAP43 silencing depressed the effect of Scu administration in promoting neuronal viability and axonal growth

**Fig. 3:**
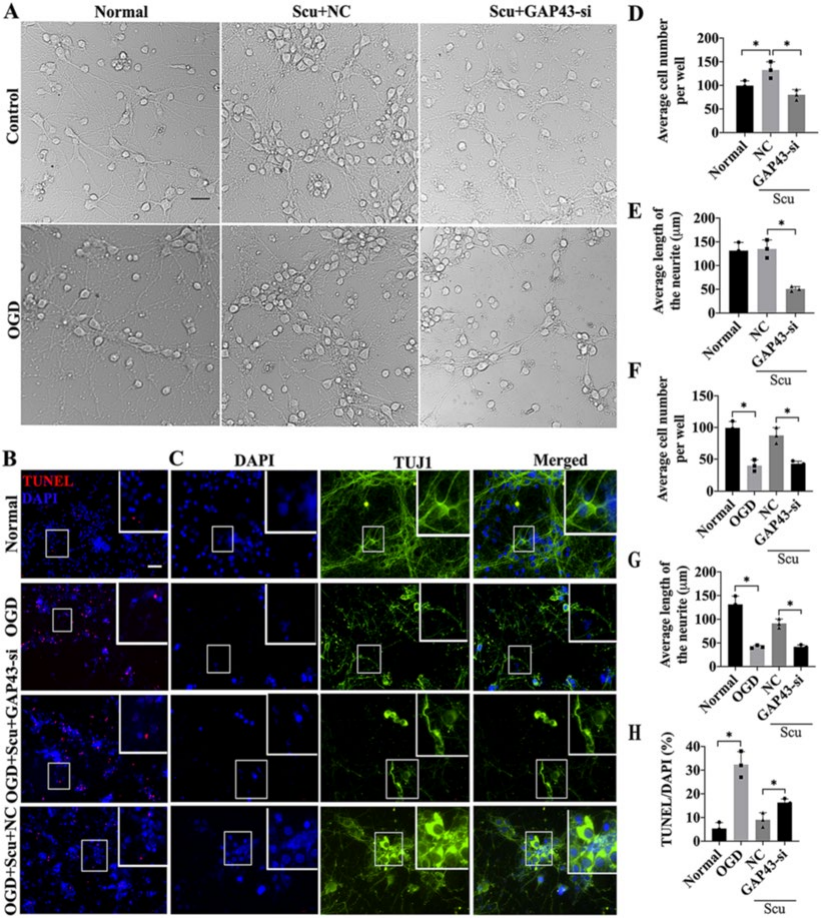
The impact of GAP43 silencing on the therapeutic efficacy of Scu treatment. **a** Light field images of neurons in the Control, Normal, Scu + NC, Scu + GAP43-si, OGD, OGD + Scu + NC, OGD + Scu + GAP43-si groups. **b** Morphological changes of neurons in the normal, OGD, OGD + Scu + GAP43-si, OGD + Scu + NC groups by TUNEL/DAPI staining. **c** Immunostaining of Tuj1 cells in the former 4 groups. **d** Cell number comparison among the normal, Scu + NC, Scu + GAP43-si groups. **e** Average length changes of neurites in aforementioned three groups. **f** Cell number comparison in the normal, OGD, OGD + Scu + NC, OGD + Scu + GAP43-si groups. **g** Average length changes of neurites among four groups above. **h** TUNEL/DAPI staining results in the normal, OGD, OGD + Scu + NC, OGD + Scu + GAP43-si groups. *OGD* Oxygen–glucose deprivation, *Scu* Scutellarin, *NC* negative control, *GAP43-si* GAP43 silencing. *p < 0.05, scale bar = 50 μm, n = 3/group

After transfection with effective siRNA sequences, the neurons were subjected to OGD and then treated with Scu. Significant difference was observed among groups in light filed images. The number of cells and the length of neurites were significantly increased in the Scu + NC group compared with that of the normal group (*p* < 0.05), while the number and length in the Scu + GAP43-si group showed notable decrease relative to that in Scu + NC group (Fig. 3a, d, e, p < 0.05). Correspondingly, immunostaining of Tuj1 and DAPI revealed that the cell number and the axonal length were markedly reduced in the OGD group in comparison to the control group, but Scu administration improved the status. However, GAP43 silencing significantly decreased the Scu-elevated cell number and the neurites length (Fig. 3c, f, g, p < 0.05). TUNEL staining was further employed to detect the cell apoptosis in the normal, OGD, OGD + Scu + NC, OGD + Scu + GAP43-si groups. The outcomes demonstrated that GAP43 depletion increased the number of apoptotic cells which were reduced by Scu treatment (Fig. 3b, g, p < 0.05). Biologically, the drug targets of Scu were involved in apoptosis-related processes and pathways indicated by network pharmacological analysis (Additional file 1: Figure S1). These results revealed that Scu administration could promote cell survival, axonal growth and decrease cell apoptosis by regulating GAP43 expression.

### Knocking out GAP43 increased infarct and cellular apoptosis of the brain in the HI rat

**Fig. 4:**
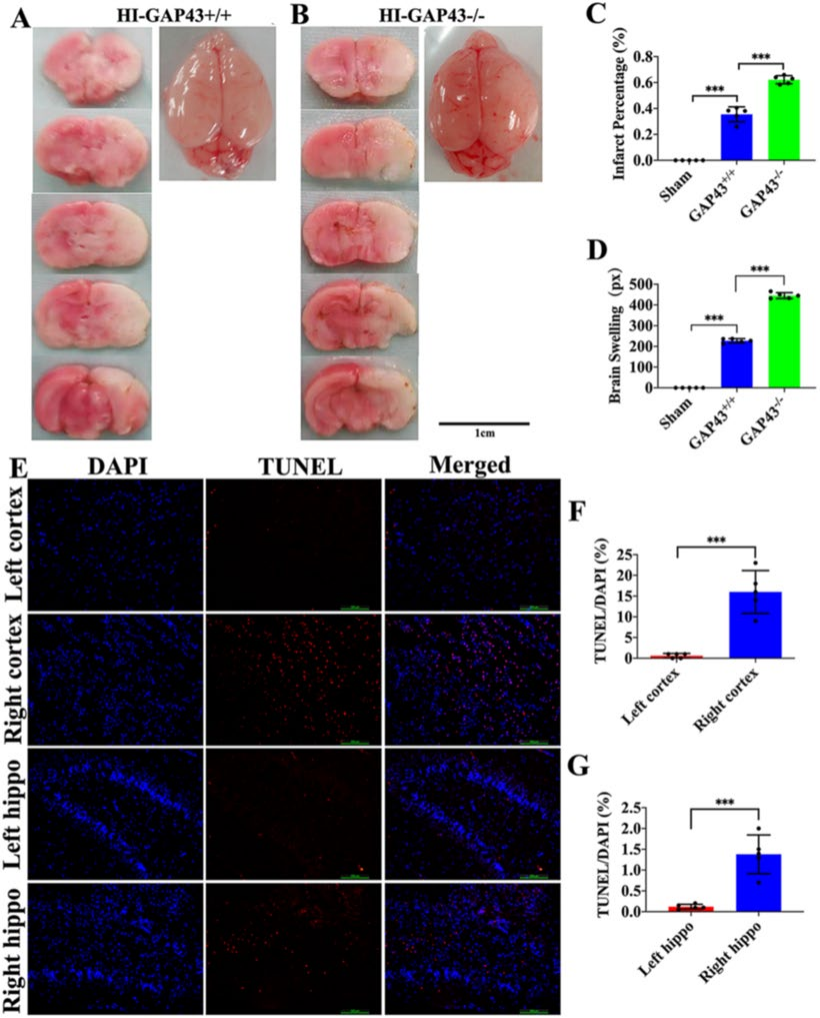
Outcomes of GAP43 knockout on cerebral damage and cell survival in HI rats. **a**, **b** The gross anatomical characteristic of rat brain surface and TTC staining slices after HI in the GAP43^+/+^ and GAP43^−/−^ groups, scale bar = 1 cm. **c** Comparison of brain infarction ratio among the sham, GAP43^+/+^, GAP43^−/−^ groups. **d** Brain swelling comparison in the above-mentioned three groups. **e** TUNEL/DAPI staining images of cortex and hippocampus in the GAP43^−/−^ rats, scale bar = 100 μm. **f** Cell apoptosis rate of the left and right cortex in the GAP43^−/−^ rats. **g** Cell apoptosis rate of the left and right hippocampus in the GAP43^−/−^ rats. *HI* hypoxia ischemia, *GAP43*^*+/+*^ wild-type rat, *GAP43*^*−/−*^ GAP43 knockout. ***p < 0.001, n = 5/group

To further validate the function of GAP43 in rats with HI injury, we established HI model using GAP43 knockout (GAP43^−/−^) rats. At 24 h after HI injury, TTC staining was applied and the outcomes showed that the cerebral ischemic area of GAP43^−/−^ rats was obviously larger than that of GAP43^+/+^ rats at 24 h post operation (Fig. 4a, b). Furthermore, the infarct ratio was higher and brain swelling area was larger in GAP43^−/−^ rats after HI injury than that of GAP43^+/+^ rats (Fig. 4c, d, p < 0.001). Moreover, TUNEL staining demonstrated higher proportion of celluar apoptosis in both the ipsilateral cortex and hippocampus of HI-GAP43^−/−^ rats than that of contralateral cortex and hippocampus in HI-GAP43^+/+^ rats (Fig. 4e, f, g, p < 0.001). These results demonstrated that the GAP43 knockout potentially aggravates the cerebral damage after HI.

### GAP43 knockout abolished Scu therapeutic effects on neurological deficits after HI

**Fig. 5:**
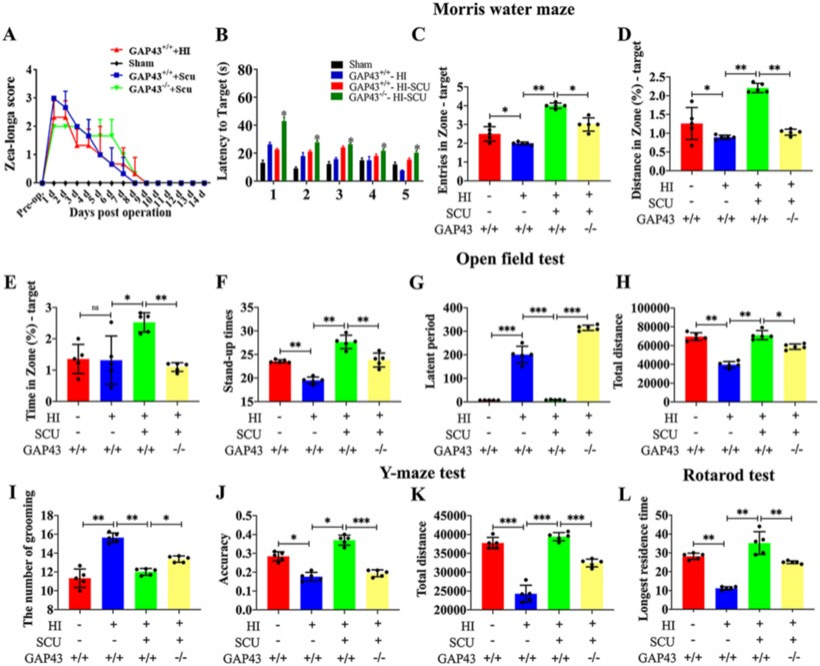
Role of GAP43 in long–term neurological function of Scu-treated HI rats. **a** Zea-longa scores of HI rats in the sham, GAP43^+/+^, GAP43^+/+^  + Scu, GAP43^−/−^ + Scu groups. **b** The latency to target in these four groups in the Morris water maze test, respectively. **c**–**e** The number of crossing original location of platform and the distance and time for crossing the original location of quadrant in the GAP43^+/+^, GAP43^+/+^  + HI, GAP43^+/+^  + HI + Scu, GAP43^−/−^ + HI + Scu groups. **f**, **i** The stand-up times and the number of grooming in the open field test in these groups. **g**, **h** The latent period and total distance in the open field test among the four groups. **j**, **k** The proportion of food arm entries and total distance finding the food arm of Y-maze in the above-mentioned groups. **l** The longest residence time in Rotarod test among these groups. *GAP43*^*+/+*^ wild-type rats; *GAP43*^*−/−*^ GAP43 knockout, *HI* hypoxia–ischemia, *Scu* Scutellarin, *d* days. *p < 0.05 vs. GAP43^+/+^  + HI + Scu, **p < 0.01 vs. GAP43^+/+^  + HI + Scu, ***p < 0.001 vs. GAP43^+/+^  + HI + Scu, n = 5/group

To further investigate the role of GAP43 in the therapeutic efficacy of Scu treatment, we constructed GAP43 knockout rats which were subjected to HI injury. Zea-longa scores test was applied to evaluate the neurological injury of neonatal rats within two weeks after HI injury. The results demonstrated that the scores in the GAP43^+/+^  + HI group were higher than that of the sham group within 9 days after operation. The scores in GAP43^+/+^  + Scu were higher than that in the GAP43^−/−^ + Scu group at 1, 2 and 3 d but they declined linearly and were lower than that in the GAP43^−/−^ + Scu group from 3 to 9 d. No obvious difference was observed in the sham group (Fig. 5a). Neurobehavioral functions were further evaluated by Morris water maze test, open field test, Y-maze test and Rotarod test at 8 weeks after HI. For Morris water maze test, the latency to target during 5 days’ training was markedly increased in GAP43^−/−^ + HI + Scu group relative to that in the GAP43^+/+^  + HI + Scu group (Fig. 5b, p < 0.05). And the number of target crossing, the percentage of distance and time in target quadrant were substantially decreased in the GAP43^+/+^  + HI group compared to sham group, except no statistical significance in time percentage between the two groups (*p* < 0.05, *p* < 0.01), but they were all significantly elevated in GAP43^+/+^  + HI + Scu group (*p* < 0.05, *p* < 0.01), which were then reversed in GAP43^−/−^ + HI + Scu group (Fig. 5c, d, e, p < 0.05, *p* < 0.01). Open field test demonstrated that stand-up times and total distance were notably decreased in GAP43^+/+^  + HI rats relative to sham group (*p* < 0.01), but augmented significantly in GAP43^+/+^  + HI + Scu group (Fig. 5f, h, p < 0.01). However, GAP43 knockout further contrarily reduced the times and distance in Scu-treated HI rats (Fig. 5f, h, p < 0.05, *p* < 0.01). In addition, the latent period and the number of grooming exhibited dramatic increase in GAP43^+/+^  + HI group in comparison with that of sham group (*p* < 0.01, *p* < 0.001), which showed reduction after Scu treatment (Fig. 5g, i, p < 0.01, *p* < 0.001). Contrarily, GAP43 knockout elevated the latent period and the number of grooming in Scu-treated groups (Fig. 5g, i, p < 0.05, *p* < 0.001). The accuracy rate of right arm entries and total distance in Y-maze test were also markedly diminished after HI injury in GAP43^+/+^ rats, which were reversely increased after in GAP43^+/+^  + HI + Scu group but further decreased in GAP43^−/−^ + HI + Scu group (Fig. 5j, k, p < 0.05, *p* < 0.001). Moreover, Rotarod test exhibited that the reduced longest residence time in GAP43^+/+^  + HI group was markedly increased in GAP43 + / +  + HI + Scu group, however, which was surprisingly shrunk after GAP43 knockout (Fig. 5l, p < 0.01). Together, GAP43 knockout significantly hindered the therapeutic effect of Scu on neonatal rats with neurological deficits induced by HI.

### Interaction of GAP43 with PTN, JAK2 and STAT3

**Fig. 6:**
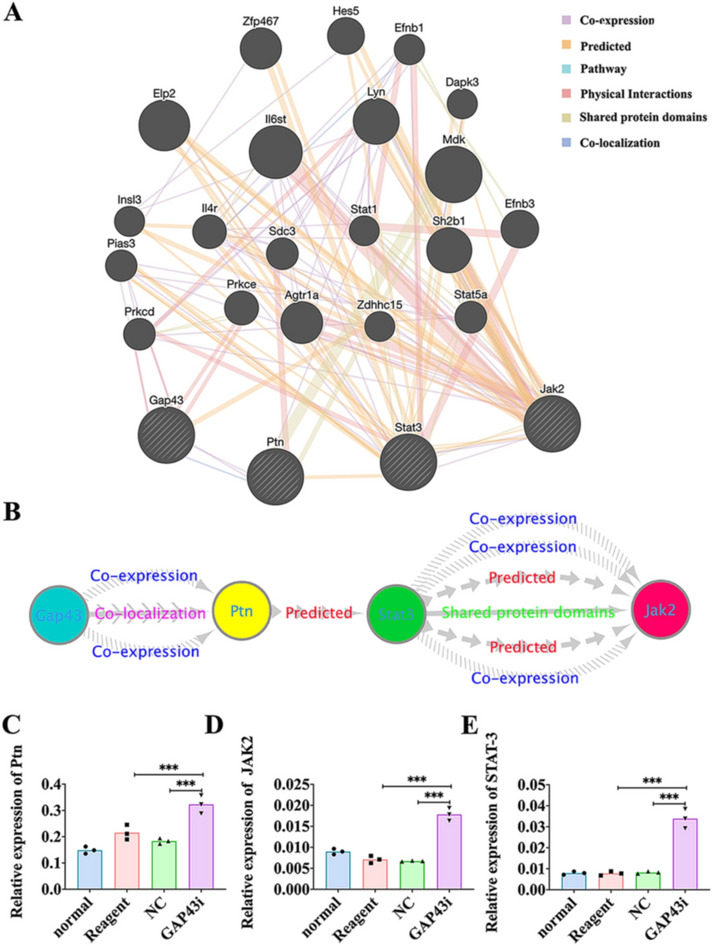
The interaction among GAP43, PTN, STAT3 and JAK2. **a** GeneMANIA analysis on genes which GAP43 were co-expressed and co-localized. **b** The mutual relation among GAP43, PTN, JAK2 and STAT3. The backward slash represents co-expression. The contiguous arrow represents co-localization. The separate arrow represents prediction. The solid line represents shared protein domains. **c**–**e** The relative expression changes of PTN, STAT3 and JAK2 after GAP43 interference in the normal, Reagent, NC, GAP43i groups, respectively. *NC* negative control, *GAP43i* GAP43 interference. Data are presented as the mean ± SD. *** *p* < 0.001, n = 3/group

Genemania analysis (http://genemania.org/) (Fig. 6a, b) revealed GAP43 was co-expressed and co-localized with PTN which predicted STAT3. Meanwhile, STAT3 was shown to co-express and share protein domains with JAK2 and STAT3 also predicted JAK2. Thus JAK2/STAT3 might be downstream activation molecules of PTN. Thus, qRT-PCR was performed to elucidate the correlation between GAP43 expression and the expression of these three factors. It was found that the expression of PTN, JAK2 and STAT3 was greatly increased after interfering GAP43 expression compared with the Reagent and NC groups (Fig. 6c–e, p < 0.001).

## Discussion

In the present study, we found that HI-induced downregulation GAP43 was rescued by Scu treatment which could promote cell viability and axonal elongation in vivo and in vitro. Functional experiments revealed that both depletion and deletion of GAP43 expression impeding the therapeutic efficacy of Scu treatment, aggravating cell apoptosis and cerebral damage, and deteriorating the cognitive and motor dysfunctions. These findings determined the crucial role of GAP43 in Scu-treated HI damage.

### Scu enhanced neuronal growth and axonal elongation, attenuated apoptosis and improved long-term HI-induced neurological deficits in vivo and in vitro

Scu has been widely studied in the field of carcinoma, for example, Burkitt lymphoma [26], tongue carcinoma [27] and hepatocellular carcinoma [28]. Scu has multiple roles including anti-inflammatory and anti-apoptotic properties and decrease blood pressure in hypertensive rats [29]. Besides, Scu could induce vasodilation [30] and exhibit heightened neuroprotective effects [31], improving the learning and memory ability of dementia rats [32, 33]. These studies are in essential agreement with our study that Scu preserved neurons, decreased apoptosis, promoted neurites outgrowth and recovered the biological function of neurons. We tested effectiveness of different doses of Scu in OGD neurons. Three μM dosages was the most effective, which can significantly promote neuronal growth and suppress neuronal apoptosis. It was observed that the GAP43 expression was up-regulated with Scu administration in OGD neurons. Besides, both the number of neurons and the length of neurites were declined and neuronal apoptosis was induced notably after down-regulation of GAP43 associated with Scu administration in HI rats. In addition, the learning and memory abilities as well as motor function of GAP43^−/−^rats were obviously decreased. However, the underlying mechanism remains unclear and needs to be further investigated.

### Downregulation and knockout of GAP43 disturbed the therapeutic efficacy of Scu in alleviating cerebral damage and neurological deficits induced by HI injury.

In the current study, we found that HI-induced downregulation of GAP43 was reversed following Scu administration, thus it’s a good candidate to study the mechanism of Scu. To exclude numerous confounding pathological elements existing in in vivo models, in vitro OGD models with GAP43 silencing were established to investigate the function of GAP43 function in the process of Scu administration. Our data showed that cell viability and axonal growth which were enhanced by Scu was declined after the depletion of GAP43, and cell apoptosis was notably induced simultaneously, indicating that GAP43 plays a crucial part in promoting axonal growth and inhibiting apoptosis. Intriguingly, the network pharmacological outcomes exhibited the targets of Scu were associated with apoptosis-related biological processes and pathways. These suggested that Scu potentially depressed cell apoptosis via upregulating GAP43. These findings were in essential agreement with previous studies reporting that GAP43 overexpression in transgenic mice has been demonstrated to promote neurite outgrowth at the adult neuromuscular junction [34]. GAP43 is of vital importance during axon growth and guidance, promoting neurite formation and inhibiting cell apoptosis.

To further validate the effects of GAP43 in attenuating long-term neurological impairments, GAP43^−/−^ rats were constructed using CRISPR/Cas-mediated genome engineering. The learning and memory abilities as well as motor function in GAP43^−/−^ rats were obviously decreased in contrast to GAP43^+/+^ rats, which was reflected by the water maze test, open field test, Y-maze test and rotarod test. The decrease in learning and memory abilities as well as motor function in GAP43^−/−^ rats correspond to the study which found that GAP43 knockout rats die early in the postnatal period and showed pronounced guidance deficits [35]. GAP43 has been known as a ‘growth’ or ‘plasticity’ protein [35], accumulating evidence revealed that low levels of GAP43 affected the recovery of brain function in rats after Scu administration and therefore further confirmed that GAP43 plays a crucial role in ameliorating long-term neurological dysfunction induced by HI injury.

### Scu exerted neuroprotective effects via GAP43-mediated signaling pathway

It was demonstrated here that GAP43 was closely associated with PTN, JAK2 and STAT3 in accordance with the results of GeneMANIA analysis. Additionally, PCR verification test showed that the levels of PTN, JAK2 and STAT3 were surprisingly elevated after induction of GAP43 silencing. GAP43 is knowingly expressed in the growth cones [36] and it could mark the axonal elongation in the process of neural development and regeneration [37]. The stimulation of GAP43 activation by Scu may stem from its activity of neurite formation and axonal regeneration by suppressing neuronal apoptosis [38]. In the previous studies, GAP43 expression was revealed in fetal brain, thalamus and prefrontal cortex [39], where it was co-expressed and co-localized with PTN [40]. A separate study has found that the mRNA levels of GAP43 were up-regulated by PTN expression in the cultured embryonic neurons, which might enhance the neurite outgrowth in the developing nervous system [41]. PTN expression is also found in human atherosclerotic plaques, and its expression in macrophages is specifically regulated by IFN-γ through the JAK–STAT1 pathway [42]. PTN, overexpressed in numerous cancers, is involved in cell transformation, growth, survival, migration and angiogenesis [43]. STAT3 signaling pathway is known to play crucial roles in tumor cells survival, and proliferation [44]. A JAK2/STAT3 signaling pathway has been previously found activated abnormally in varieties of tumor tissues, which exerts great impact on tumor progression [45]. In this study, we found that Scu treatment promoted GAP43 expression in OGD neurons, coinciding with the reduction of PTN, STAT3 and JAK2 expression. As these genes was upregulated after GAP43 silencing in HI rats with Scu administration, thus neurological dysfunction induced by HI injury correlated with GAP43-mediated high levels of PTN, STAT3 and JAK2. This phenomenon might be explained by the fact that GAP43 enhances the action of G-protein-coupled receptors [46], which are negatively affected after interference with GAP43, resulting in certain signal transduction pathways affecting the expression of PTN, JAK and STAT3. In the present study, Scu may be closely associated with GAP43, PTN, JAK2 and STAT3. However, the in-depth mechanisms involving GAP43 are very complex, and we are using dual-luciferase reporter assay and selective activation and inhibition experiments to elucidate the role of GAP43-related pathways in Scu therapy for the development of HI.

## Conclusions

Taken together, Scu treatment could improve cell viability, and ameliorate cell apoptosis and long-term neurological deficits after HI injury via upregulating GAP43 expression. Hence, the present study might provide novel molecular mechanisms and therapeutic efficacy for the clinical treatment of HIE.

## Supplementary Information

**Additional file 1: Figure S1.** The GO terms and KEGG pathway analysis of drug targets of Scu. (A-C) GO enrichment analysis for drug targets of Scu, including (**A**) BP, (**B**) MF and (**C**) CC, respectively. **(D)** KEGG enrichment analysis for drug targets of Scu. GO, gene ontology; KEGG, Kyoto Encyclopedia of Genes and Genomes; BP, biological processes; MF, molecular function; CC, cellular component.

## Data Availability

The dataset(s) supporting the conclusions of this article is(are) included within the article [and its additional file(s)].

## Abbreviations

HIE: Hypoxic-ischemic encephalopathy
HI: Hypoxia–ischemia
OGD: Oxygen–glucose deprivation
RT-qPCR: Real-time quantitative polymerase chain reaction
TUNEL: Terminal-deoxynucleotidyl Transferase Mediated Nick End Labeling
DAPI: 4ʹ,6-Diamidino-2-phenylindole
Scu: Scutellarin
F1: Fragment 1
F2: Fragment 2
GAP43i: GAP43 silencing
GAP43^+^^/^^+^: Wild-type rat
GAP43^−^^/^^−^: GAP43 knockout
Min: Minutes
NC: Negative control
d: Days

## References

[1] Finer NN (1981). Hypoxic-ischemic encephalopathy in term neonates: perinatal factors and outcome. J Pediatr.

[2] Vannucci RC, Perlman JM (1997). Interventions for perinatal hypoxic-ischemic encephalopathy. Pediatrics.

[3] Kawarai Y (2018). Progesterone as a postnatal prophylactic agent for encephalopathy caused by prenatal hypoxic ischemic insult. Endocrinology.

[4] Guillet R, Edwards A, Thoresen M (2012). Seven-to eight -vear follow-up of the coolcap trial of head cooling forneonatal encephalopathy. Pediatric.

[5] Ogihara T (2003). Non-protein-bound transition metals and hydroxyl radical generation in cerebrospinal fluid of newborn infants with hypoxic ischemic encephalopathy. Pediatr Res.

[6] Fitzgerald MP, Kessler SK, Abend NS (2017). Early discontinuation of antiseizure medications in neonates with hypoxic–ischemic encephalopathy. Epilepsia.

[7] Gonzalesportillo GS (2014). Stem cell therapy for neonatal hypoxic-ischemic encephalopathy. Front Neurol.

[8] Adam F (2013). Gentamicin pharmacokinetics and dosing in neonates with hypoxic ischemic encephalopathy receiving hypothermia. Pharmacotherapy.

[9] Lin L (2007). Protective effects of scutellarin and breviscapine on brain and heart ischemia in rats. J Cardiovasc Pharmacol.

[10] Liu H (2003). Protective effects of scutellarin on superoxide-induced oxidative stress in rat cortical synaptosomes. Acta Pharmacol Si.

[11] Tang H (2014). Neuroprotective effects of scutellarin and scutellarein on repeatedly cerebral ischemia-reperfusion in rats. Pharmacol Biochem Behav.

[12] Pan Z (2011). Scutellarin alleviates interstitial fibrosis and cardiac dysfunction of infarct rats by inhibiting TGFβ1 expression and activation of p38-MAPK and ERK1/2. Br J Pharmacol.

[13] Fang M (2015). Scutellarin regulates microglia-mediated TNC1 astrocytic reaction and astrogliosis in cerebral ischemia in the adult rats. BMC Neurosci.

[14] Guo H (2011). Neuroprotective effects of scutellarin against hypoxic-ischemic-induced cerebral injury via augmentation of antioxidant defense capacity. Chin J Physiol.

[15] Zhang HF (2009). Protective effects of scutellarin against cerebral ischemia in rats: evidence for inhibition of the apoptosis-inducing factor pathway. Planta Med.

[16] Verge VM (1990). Correlation between GAP43 and nerve growth factor receptors in rat sensory neurons. J Neurosci.

[17] Meiri KF, Pfenninger KH, Willard MB (1986). Growth-associated protein, GAP-43, a polypeptide that is induced when neurons extend axons, is a component of growth cones and corresponds to pp46, a major polypeptide of a subcellular fraction enriched in growth cones. Proc Natl Acad Sci USA.

[18] Benowitz LI, Routtenberg A (1997). GAP-43: an intrinsic determinant of neuronal development and plasticity. Trends Neurosci.

[19] Skarnes WC (2011). A conditional knockout resource for the genome-wide study of mouse gene function. Nature.

[20] Singhal S (2005). Prognostic implications of cell cycle, apoptosis, and angiogenesis biomarkers in non-small cell lung cancer: a review. Clin Cancer Res.

[21] Gorup D (2015). Increased expression and colocalization of GAP43 and CASP3 after brain ischemic lesion in mouse. Neurosci Lett.

[22] Xiong LL (2020). Vi4-miR-185-5p-Igfbp3 network protects the brain from neonatal hypoxic ischemic injury via promoting neuron survival and suppressing the cell apoptosis. Front Cell Dev Biol.

[23] Ferrari DC, Nesic OB, Perez-Polo JR (2010). Oxygen resuscitation does not ameliorate neonatal hypoxia/ischemia-induced cerebral edema. J Neurosci Res.

[24] Zhang ZB (2019). miRNA-7a-2-3p inhibits neuronal apoptosis in oxygen-glucose deprivation (OGD) model. Front Neurosci.

[25] Franz M (2018). GeneMANIA update 2018. Nucleic Acids Res.

[26] Feng Y (2012). Novel function of scutellarin in inhibiting cell proliferation and inducing cell apoptosis of human Burkitt lymphoma Namalwa cells. Leuk Lymphoma.

[27] Li H (2010). Scutellarin inhibits cell migration by regulating production of αvβ6 integrin and E-cadherin in human tongue cancer cells. Oncol Rep.

[28] Xu H, Zhang S (2013). Scutellarin-induced apoptosis in HepG2 hepatocellular carcinoma cells via a STAT3 pathway. Phytother Res PTR.

[29] Chen X (2013). Scutellarin attenuates hypertension-induced expression of brain Toll-like receptor 4/nuclear factor kappa B. Mediat Inflamm.

[30] Chen YJ (2015). Scutellarin attenuates endothelium-dependent aasodilation impairment induced by hypoxia reoxygenation, through regulating the PKG signaling pathway in rat coronary artery. Chin J Nat Med.

[31] Tang H (2015). Investigation on the interactions of scutellarin and scutellarein with bovine serum albumin using spectroscopic and molecular docking techniques. Arch Pharm Res.

[32] Guo LL, Wang YL, Huang Y (2011). Effect of scutellarin on expressions of nicotinic acetylcholine receptor protein and mRNA in the brains of dementia rats. Zhongguo Zhong xi yi jie he za zhi Zhongguo Zhongxiyi jiehe zazhi Chin J Integr Trad Western Med.

[33] Guo L, Guan Z, Wang Y (2011). Scutellarin protects against Aβ-induced learning and memory deficits in rats: involvement of nicotinic acetylcholine receptors and cholinesterase. Acta Pharmacol Sin.

[34] Caroni P, Aigner L, Schneider C (1997). Intrinsic neuronal determinants locally regulate extrasynaptic and synaptic growth at the adult neuromuscular junction. J Cell Biol.

[35] Yiping S (2002). Growth-associated protein-43 is required for commissural axon guidance in the developing vertebrate nervous system. J Neurosci.

[36] Skene JH (1986). A protein induced during nerve growth (GAP-43) is a major component of growth-cone membranes. Science.

[37] Oestreicher AB (1997). B-50, the growth associated protein-43: modulation of cell morphology and communication in the nervous system. Prog Neurobiol.

[38] Hung CC (2016). Astrocytic GAP43 Induced by the TLR4/NF-κB/STAT3 Axis Attenuates Astrogliosis-Mediated Microglial Activation and Neurotoxicity. J Neurosci.

[39] Benowitz LI (1989). Localization of the growth-associated phophoprotein GAP-43 (B-50, F1) in the human cerebral cortex. J Neurosci.

[40] Walker JR (2004). Applications of a rat multiple tissue gene expression data set. Genome Res.

[41] Yanagisawa H (2010). Pleiotrophin induces neurite outgrowth and up-regulates growth-associated protein (GAP)-43 mRNA through the ALK/GSK3beta/beta-catenin signaling in developing mouse neurons. Neurosci Res.

[42] Li F (2010). Pleiotrophin (PTN) is expressed in vascularized human atherosclerotic plaques: IFN-{gamma}/JAK/STAT1 signaling is critical for the expression of PTN in macrophages. FASEB J.

[43] Reiff T (2011). Midkine and Alk signaling in sympathetic neuron proliferation and neuroblastoma predisposition. Development.

[44] Sandur SK (2010). 5-hydroxy-2-methyl-1,4-naphthoquinone, a vitamin K3 analogue, suppresses STAT3 activation pathway through induction of protein tyrosine phosphatase, SHP-1: potential role in chemosensitization. Mol Cancer Res.

[45] Wang G (2018). Crocin promotes apoptosis of human skin cancer cells by inhibiting the JAK/STAT pathway. Exp Ther Med.

[46] Strittmatter SM (1993). GAP-43 augments G protein-coupled receptor transduction in *Xenopus laevis* oocytes. Proc Natl Acad Sci U S A.

