# Supplementary figures and images for: Scutellarin ameliorates neonatal hypoxic-ischemic encephalopathy associated with GAP43-dependent signaling pathway
Source: Chin Med. 2021 Oct 18;16:105. doi: 10.1186/s13020-021-00517-z (PMC8524967; doi:10.1186/s13020-021-00517-z)

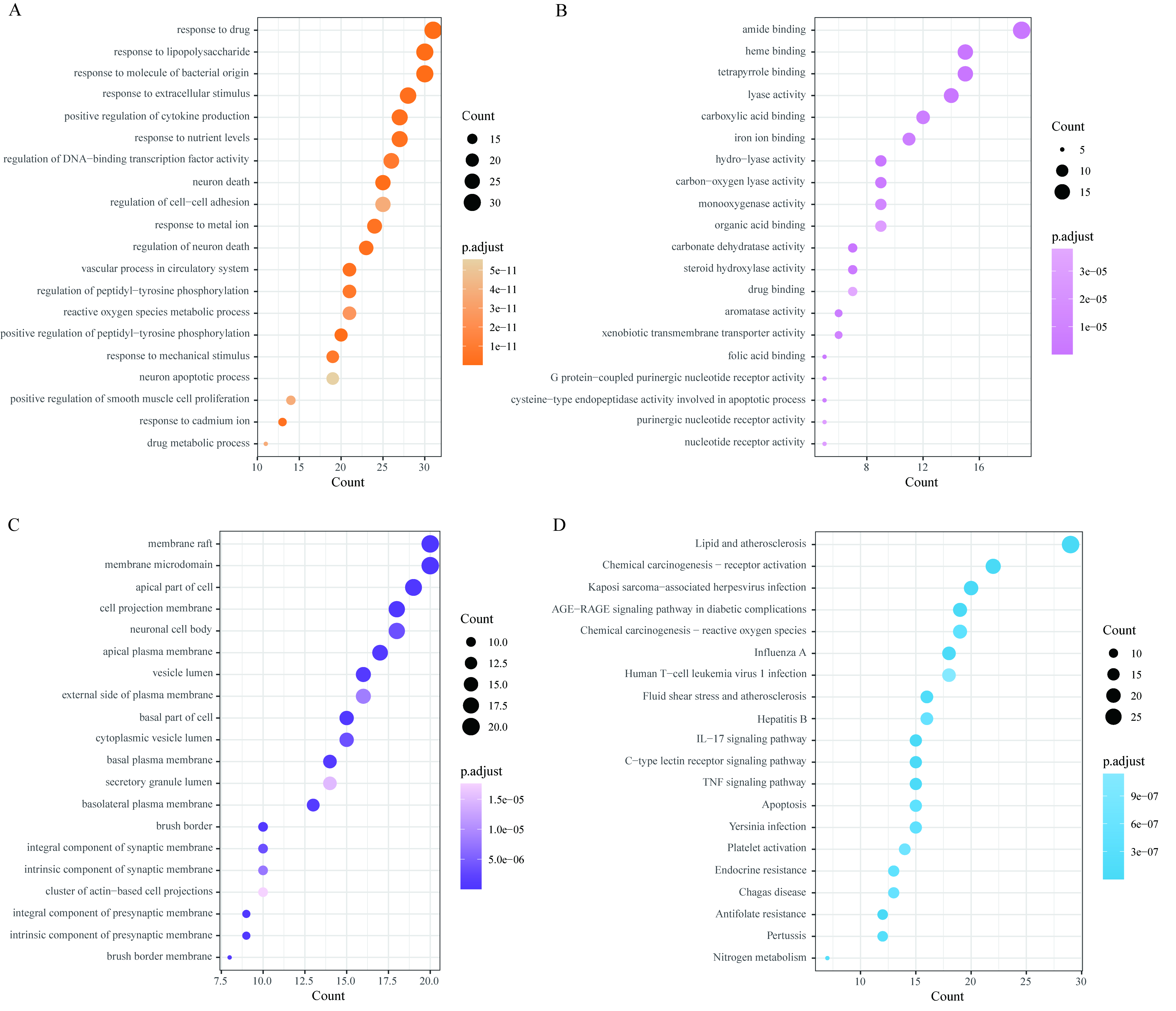

Supplement: Supplementary file 1 — Additional file 1: Figure S1. The GO terms and KEGG pathway analysis of drug targets of Scu. (A-C) GO enrichment analysis for drug targets of Scu, including (A) BP, (B) MF and (C) CC, respectively. (D) KEGG enrichment analysis for drug targets of Scu. GO, gene ontology; KEGG, Kyoto Encyclopedia of Genes and Genomes; BP, biological processes; MF, molecular function; CC, cellular component. [file 13020_2021_517_MOESM1_ESM.tif]
